# 

**Published:** 1880-01

**Authors:** 


### INDEX.

| | PAGE |
| --- | --- |
| Abscesses, renal, in a cow | 101 |
| Abortion, epidemic in cattle | 34 |
| Acid, salicylic | 181–244 |
| Agriculture, report of commissioner of—review | 157 |
| Anatomy of the horse's hoof | 24–73 |
| Animals, domestic, leucocythaemia in | 35 |
| Animals, depraved taste in | 242 |
| Animals, rabies in | 185 |
| Animal tuberculosis | 136 |
| Ant, the harvesting | 247 |
| Antelope, brain, cysticercus of | 33 |
| Anthelmintic, a new | 171 |
| Anthrax and the germ-theory of disease | 96 |
| pathological course of | 38 |
| Anus, constriction of, in a bull | 109 |
| Aorta, glands on the side of, in a dog | 103 |
| Archives, objects and scope of the | 26 |
| Arsenite of copper, acute case of poisoning by | 238 |
| Articulations, wounds of, in the horse | 108 |
| Ascarides, death of a dog, caused by | 240 |
| Asymmetry, embryonic | 104 |
| Atrophy, localized muscular | 10 |
| | |
| Baboon, cysticerci in brain of | 33 |
| Bees, carnivorous habits in | 176 |
| Berns, G. H.—A case of acute articular rheumatism | 98 |
| colic in horses | 149 |

Berns, rupture of the gastrocnemius and tendons of the flexor pedis perforatus muscles 163
tympanitis 218
Birds, aquatic, domestication of 181
fondness for music in 248
Bladder, urinary, in a lioness—rupture of 31
Blood 57
filaria, treatment of 109
Blood stains 250
Bovine, wild, pleuro-pneumonia in the 36
Box, for pamphlets 184
Brain of the antelope, cystercicus in 33
of baboon, cysticerci in 33
of the iguana 103
of the pteropus 103
Bronchitis, parasitic, in dogs 34
Bull, constriction of the anus in 109

Canines, influence of taxation on 112
spaying the female 153
Calculus in urethra 165
Calf, monstrosity in 175
Capillary blood vessels, contractility of 172
Cattle, atrophy of pancreas in 39
epidemic abortion in 34
fungoid disease in 39
infected 182
inoculation of, in pleuro-pneumonia 106
lung plague of 19
Camels, veterinary experience with 34
Camelidæ, observations on 207
Canaries, color of plumage 246
Carbuncle and earthworms 246
Carcinoma of the pancreas and omentum in an oclos-feli-pardalis 237
Caries of the os coronary and os pedis 234
Castration of the dog 87
Cats as post carriers 247
diphtheria spread by 241

Cells, growth as a function of ............ 183
Chicken cholera ............ 109
monstrosities of ova in ............ 35
Colon, displacement of, in a horse ............ 106
Columbia veterinary college, opening exercises of 1879 ............ 47
commencement of ............ 79
opening exercises of 1880 ............ 252
Comments on an act to regulate the practice of veterinary medicine ............ 91
Conklin, Wm. A.—habits and treatment of the felidæ in captivity 132
observations on the camelidæ ............ 207
"Corn" in the equine foot ............ 79
Cow, renal abscess in ............ 101
effect of want of water in ............ 184
inversion of uteris in ............ 166
Cressy, Noah.—diseased meat and its consequences upon our health and happiness ............ 193
Curare ............ 175
Cysticercus of brain in antelope ............ 33
in brain of baboon ............ 33

DeVoe, Thos. F.—food fishes and their diseases ............ 223
Disease, contagious, among cattle, prevention of ............ 94
germ theory of, and anthrax ............ 96
Dog, aorta of, glands on the side of ............ 103
castration of the ............ 87
death of, caused by ascarides ............ 240
heart, filaria in ............ 23
heart of a setter ............ 244
parasitic bronchitis in ............ 34
Dowd, D. E.—cutting or interfering in horses—how remedied 155
Dura Mater, tumor of, in a horse ............ 31

Earl, H. E —heart filaria in a dog ............ 23
Earthworms and carbuncle ............ 246
Elephant, placenta of ............ 178
Emphysema, contagiosum ............ 38
Epizootic in venezuela ............ 33
Epidemic in champlain county ............ 252

Epizootic fever ........ 251
Equine typhus ........ 37
Ethyl, bromide of ........ 243
Eucalyptus globulus ........ 242
Extremities, paralysis of posterior ........ 235

Felidæ in captivity, habits and treatment of ........ 132
Fever, epizootic ........ 257
Fibroma, four cases of ........ 168
Filaria in the blood, treatment of ........ 109
Fish, breeding ........ 249
their diseases ........ 223
Flexor pedis perforatus, rupture of ........ 163
Fluid, a new preservative ........ 178
Foot, horse's, anatomy of ........ 24–73
of sheep, its care and neglect ........ 243
Fowl, hallucination in ........ 100
Fox, meningitis, metastatic, in a ........ 33
Fracture of seventh rib ........ 234

Garden, zoological, of central park ........ 182
Gastrocnemius, rupture of ........ 163
Germ disease theory ........ 176
Glanders, concealed, in a horse ........ 105
diagnosis of ........ 36
and pyaemia, the relation between ........ 109
Glaucoma in a panther ........ 56
Goose, cause of the white ........ 248
Gout, a new theory of ........ 177
Greenfield, W. S.—recent investigations into the pathology of infective and contagious diseases ........ 142–210

Hadden, Alexander.—castration of the dog, desirability, etc ........ 87
Haematuria in the roebuck ........ 35
Hæmoglobinuria ........ 240
toxaemia, in a horse ........ 110
Hair, horse without ........ 113
Hallucination in a fowl ........ 100

Hamill, James.—peculiar deformity in a horse, due to localized muscular atrophy........ 10
On the affection termed "corn" in the equine foot........ 79
Hawkins, Thos. H.—vivisection, lecture on........ 47
Heath, Allen S.—animal tuberculosis, its transmissibility to the human subject through diseased meat and milk........ 136
Health, new state board of........ 183
Heart, filaria, in a dog........ 23
of a setter dog........ 244
Hegeman, J. Aycrigg, and Porter, W. H.—pathological changes in lung plague of cattle........ 14
Hens, habit of and cure........ 243
Hind-quarters of the horse, paralysis of........ 3
Hippopotamus, pneumonia, catarrhal, in a........ 32
Hog cholera........ 184
Horse, absence of kidney in........ 106
bedding, new materials for........ 56
colic in........ 149
cutting or interfering, how remedied........ 155
concealed glanders in a........ 105
danger of contagion from........ 244
feeding of........ 245
deformity in........ 10
fleshmeal for........ 112
danger of laryngeal insufflation in........ 112
displacement of the colon in........ 106
haemoglobinuria, toxaemic, in........ 110
hairless........ 113
intestinal torsion in........ 106
notes on........ 248
paralysis of the hind-quarters of........ 3
pyaemia, embolic, of........ 32
retina, detachment of........ 34
stomatitis of the........ 35
the American........ 115
tumor of dura mater in........ 31
wounds of the articulations........ 108
Humor, vitreous, regeneration of........ 177

Hybrids, fertile ........ 247
Hydrophobia, pathology of the nerve-centres in ........ 36
transmitted from man to the rabbit ........ 108

Iguana, the brain of the ........ 103
Insufflation, danger of in horses ........ 112
Insecticide, a new ........ 245
Interfering in horses, how remedied ........ 155
Investigations, pathological, of infective and contagious diseases ........ 142–210
Island of reil, in the porpoise ........ 41

Kidney, absence of in a horse ........ 106

Law, Prof. James.—notice of report by ........ 311
book by ........ 45
Loring Geo. B.—the American horse ........ 115
Leucocythaemia in domestic animals ........ 35
Lioness, rupture of urinary bladder of ........ 41

Mammalia and sauropsida, demarcation between ........ 103
Mare, inversion of uterus in ........ 239
McLaughlin, John A. Jr.—some new points in the anatomy of the horse's foot ........ 24–73
Meat, diseased, etc ........ 192
Meningitis, metastatic, in a fox ........ 33
tuberculous, in a sun-bear ........ 104
Meyer, Chas. A.—extension of ring-worm ........ 102
multiple renal abscesses in a cow ........ 101
the symptomatic significance of apparent paralysis of the hind-quarters of the horse ........ 3
Monkey, pachy-meningitis in ........ 238
Monstrosities of ova in chickens ........ 35
paternal influence in the production of ........ 34
Milk, diseased, evil effects of ........ 39
Muscles, sub-lumbar, traumatism of ........ 174

National bureau of veterinary inspection ........ 28–55
News items ........ 250

Omentum, carcinoma of ........ 237
Operations, questionable ........ 225
Organisms, microscopic, etc ........ 174
Organisms, microscopic, their relations to disease ........ 110
Os coronary, caries of ........ 234
Os pedis, caries of ........ 234

Pancreas, atrophy of, in cattle ........ 39
carcinoma of ........ 237
Panther, glaucoma in ........ 56
Pachy-meningitis in a monkey ........ 238
Parasites of a sunfish ........ 32
Paralysis of the hind-quarters of a horse ........ 3
Peritonitis, acute suppurative, in a sea-lioness ........ 31
Pharyngo-laryngitis, complicated with œdema glottidis ........ 166
Pig-typhoid, pathology of ........ 36
Placenta elephas ........ 178
Plague, lung, of cattle ........ 414
lung, extent of ........ 184
Pleuro-pneumonia of cattle ........ 14
epizootic, pathological histology of ........ 109
inoculation of, in cattle ........ 106
in the wild bovine ........ 36
Pneumonia, catarrhal, in a hippopotamus ........ 32
Poisoning by paris green ........ 238
Porpoise, island of reil in the ........ 41
Porter, W. H., and Hegeman, J. Aycrigg. — pathological changes in the lung plague of cattle ........ 14
Practice of veterinary medicine, an act to regulate ........ 91
Prevention of trichinosis in swine ........ 35
Pteropus, the brain of ........ 103
Pyaemia and glanders, the relation between ........ 109
embolic in a horse ........ 32

Quinia, hypodermic use of ........ 180

Rabies, inoculation of ........ 181
in the lower animals ........ 185
Rachitis in pigs ........ 36

Retina, detachments of, in horses........ 34
Reviews, department of agriculture, No. XII., 1879........ 44
the lung plague of cattle........ 45
report of commissioners of agriculture........ 157
of Prof. James Law, of Ithaca, N. Y., bulletin national board of health, Vol. II., No. IV........ 231
Rheumatism, acute, articular........ 98–265
Rhinoceros, an antediluvian........ 247
Rib, seventh, fracture of........ 234
Rinderpest in Germany........ 37
Ringworm, extension of........ 102
Roebuck, haematuria in the........ 35
Ruminants, domestication of........ 181
Rupture of gastrocnemius........ 163

Salicine, effects of, in rheumatism........ 165
Satterthwaite, Thos. E.—some of the recent contributions to the literature of comparative medicine... 185
the blood........ 57
Sauropsida and mammalia, demarcation between........ 103
Scabies........ 175
Sea-Lioness, peritonitis acute suppurative in........ 31
Sea-Lion, the nervous system of........ 41
Seguin, E. C.—an instance of hallucination in a fowl........ 100
Sex, production at will........ 172
in utero, determination of........ 172
Sheep's foot, care and neglect of........ 243
killed by dogs........ 245
rot........ 173
Smith, Quintius C—treatment of inversion of the uterus in cows........ 166
Society, a new veterinary........ 113
Soula, William.—a case of caries of the os coronary and os pedis, with fracture of seventh rib........ 234
two cases of partial paralysis of the posterior extremities........ 235
Spaying the female canine........ 153
Spitzka, E. C.—anatomical notes........ 41

Spermatozoa, structure of ........ 421
Stomatitis of the horse ........ 35
Sun-bear, tuberculous meningitis in ........ 104
Sunfish, parasites of ........ 32
Suppositories, gelatine ........ 180
Swine plague ........ 171
 prevention of trichinosis in ........ 35
 rachitis in ........ 36

Tapeworm, development of ........ 34
Taste, depraved in animals ........ 242
Taxation, influence of on canines ........ 112
Thompson N. F.—notes on four cases of fibroma ........ 168
 questionable operations ........ 225
Torsion, intestinal in the horse ........ 106
Trichinosis in swine, prevention of ........ 35
Trichinosis ........ 105
Tuberculosis, animal ........ 136
Tumor of the dura mater in a horse ........ 31
Typhus, equine ........ 37
Tympanitis ........ 218

Urethra, friable calculus in ........ 165

Uterus, inversion of in cows ........ 166

 a mare ........ 239

Veterinary bill, failure of ........ 157

 homœopathy ........ 113

 inspection, national bureau of ........ 28–55

Venezuela, epizootic in ........ 33

Vivisection ........ 228

 does it pay? ........ 160

Walton, F.—acute rheumatism treated with salicine ........ 165
Wool-sorter's disease ........ 171–240
Worm of the kidney ........ 180

